# Production of an Enzymatic Extract From *Aspergillus oryzae* DIA-MF to Improve the Fructooligosaccharides Profile of Aguamiel

**DOI:** 10.3389/fnut.2019.00015

**Published:** 2019-02-21

**Authors:** Brian Picazo, Adriana C. Flores-Gallegos, Anna Ilina, Rosa María Rodríguez-Jasso, Cristóbal N. Aguilar

**Affiliations:** Group of Bioprocesses and Bioproducts, Food Research Department, School of Chemistry, Universidad Autónoma de Coahuila, Saltillo, Mexico

**Keywords:** agavins, antioxidant, fructooligosaccharides, fructosyltransferase, inulinase, prebiotic

## Abstract

Aguamiel is a natural sap produced by some species of agave plants, such as *Agave salmiana, A. atrovirens*, or *A. angustifolia*. It is a product with a high concentration of fructose, glucose or sucrose, although its composition may vary depending on the season in which it is produced, and may also contain agave fructans (or agavins) or fructooligosaccharides (FOS). It has been reported that FOS can be produced by enzymes that act on sucrose or inulin, transfructosylating or hydrolyzing these materials, respectively. Due to the sugar content in aguamiel, the application of an enzymatic complex produced by *Aspergillus oryzae* DIA MF was carried out. This complex was characterized by 1-D electrophoresis SDS-PAGE, and its transfructosylation and hydrolysis activities were determined by HPLC. In order to determine the conditions at which the concentration of FOS in this beverage increased, kinetics were carried out at different temperatures (30, 50, and 70°C) and times (0, 1, 2, 3, 4, 5, 10, and 15 h). Finally, the antioxidant and prebiotic activities were evaluated. FOS concentration in aguamiel was increased from 1.61 ± 0.08 to 31.01 ± 3.42 g/ L after 10 h reaction at 30°C applying 10% enzymatic fraction-substrate (v/v). Antioxidant activity was highly increased (34.81–116.46 mg/eq Trolox in DPPH assay and 42.65 to 298.86 mg/eq Trolox in FRAP assay) and growth of probiotic bacteria was higher in aguamiel after the enzymatic treatment. In conclusion, after the application of the enzymatic treatment, aguamiel was enriched with FOS which improved antioxidant and prebiotic properties, so it can be used as a functional food.

## Introduction

Fructooligosaccharides (FOS) are oligosaccharides composed of fructose monomers linked to a glucose with a polymerization degree (DP) from three to ten; these oligosaccharides have β-2,1 or β-2,6 linkages in its structure. If the DP goes >10 these polysaccharides belong to the fructans group, and depending on its structure, they are named differently: inulin if the fructan structure has β-2,1 linkages, levan if it has β-2,6 linkages and, a fructan with both linkages in its structure is known as agavin (1).

FOS can provide different effects on human health. Due to the linkages they have, FOS can be eaten and reach the intestine intact because the digestive system cannot metabolize them; once they reach the intestine, the microbiota uses them as a substrate, classifying them as prebiotics (2). After these compounds are used by the microbiota as a substrate, bacteria like *Lactobacillus* or *Bifidobacterium* produce compounds to regulate cholesterol and triglycerides, increasing the mineral absorption in the intestine, and to regulate the production of diverse cytokines and immunoglobulin A, reducing the risk of developing colon cancer. It has also been reported that some FOS from the grain of *Coix lachryma-jobi* Linn can bring antioxidant activity 0.97-fold higher than of vitamin C (2–6).

FOS can be found in natural materials (e.g., onions, bananas, chicory), but its concentration is very low and by different extraction methods, the yield can be 0.009 g of FOS/ g of raw material (7). An alternative in FOS production is the employment of biotechnological processes. Some fungi and bacteria are able to produce enzymes with FOS production capacity, but fungi are mainly used due to their capacity to produce extracellular enzymes, making the production in culture media easier. Fungi can produce FOS by different enzymatic mechanisms: fructosyltransferases, which form FOS from sucrose by transfructosylation (8); β-fructofuranosidases with hydrolytic and fructosyltransferase activity, being the hydrolytic activity preponderant during transfructosylating (9); and inulinases, enzymes that hydrolyze inulin, producing fructose monomers (exoinulinase) or/and producing shorter inulin chains or FOS (endoinulinase) (10).

Infant formulas, pastry, confectionary, beverages, other food products and food supplements have been enriched with FOS. Nevertheless, the suggested minimum daily effective intake required to produce a beneficial health effect is 3–8 g FOS/portion of product (11). Agave plants can produce an exudate denominated aguamiel. Aguamiel is a liquid product rich in sugars, which are mainly composed of fructose, sucrose, FOS, and agavins; but, it also contains glucose and some maltooligosaccharides (12, 13). The concentration of FOS in aguamiel is low, but it's a material with great conditions before use as a product enriched with FOS, it has high concentrations of sucrose and agavins used to be hydrolyzed to produce more FOS. Also, aguamiel is a cheap product with a price lower than $1 dollar and in its composition is made up of saponins, minerals and essential amino acids, along with the sugars it contains (14–16). The aim of this work was to increase the FOS concentration in aguamiel, by using an enzymatic complex produced by *Aspergillus oryzae* DIA-MF, to improve its antioxidant and prebiotic properties.

## Methodology

### Reagent and Standards

FOS standards 1-kestose (GF2), 1-nystose (GF3), and 1F-fructofuranosylnystose (GF4) were purchased from Wako Pure Chemical Industries, Ltd. (Japan Company). Fructose (F), glucose (G), and sucrose (S) standards were obtained from Sigma Aldrich (St. Louis, MO, USA). A Trolox standard was obtained from Sigma Aldrich (St. Louis, MO, USA). Aguamiel was obtained from “Ejido Las Mangas” (long. −101.102500, Lat. 24.906111), Saltillo, Coahuila, Mexico from *Agave salmiana* in December 2017. It was sterilized by membrane filtration (0.45 μm) before its use. Orafti HSI® was obtained from Beneo Company (Belgium). Agavins were obtained from Megafarma® (Durango, Mexico). Protein marker Prestained SDS-PAGE standard #1610318 was obtained from Bio-Rad (Hercules, CA, USA). Silver BULLit™ silver stain kit was obtained from AMRESCO (Radnor, PA, USA).

### Evaluation and Selection of the Crude Enzymatic Extract

Three different carbon sources (Orafti HSI® and Agavins at 60 and 120 g/L, and aguamiel) were used to produce the enzymatic extracts which were applied to aguamiel to evaluate the increase in FOS concentration. These enzymatic extracts were produced by *A. oryzae* DIA-MF at 0, 6, 12, 18, 24, 30, 36, and 48 h of fermentation, giving a total of 40 crude enzymatic extracts. For each extract the enzymatic activity was evaluated over aguamiel as follows: in 2 mL Eppendorf tubes 900 μL of aguamiel were deposited, 100 μL of extract were added to aguamiel and incubated at 30°C for 20 min. The reaction was stopped at 100°C for 3 min. All samples were filtered through 0.45 μm nylon membranes. Samples were analyzed by high-performance liquid chromatography (HPLC, Perkin Elmer Series 200) using a Prevail Carbohydrate ES Column (5 μm, 250 × 4.6 mm, Grace) at 30°C. A mixture of acetonitrile/water 70:30 (v/v) and NH_4_OH 0.04% was used as a mobile phase at a flow rate of 1 mL/min with a pressure of 1,700 psi. A refractive index detector (RID) was operated at 35°C. The response of the RID was recorded and integrated using the TOTALCHROM WS V6.3 software. The quantification of FOS in samples was determined by using standards curves made with different known concentrations (17). The extract that showed the greatest increase in FOS concentration in aguamiel was selected for further steps.

### Fractioning of the Selected Enzymatic Extract

The selected extract was fractioned into four. The fractioning was made by a successive filtration with different membranes. The first membrane used to microfilter was a 450 kDa cellulose membrane in a Millipore vacuum glass system. After microfiltration, the extract was ultrafiltered in an Amicon Stirred Cell® (Millipore) in a nitrogen atmosphere, thought 300, 100, and 30 kDa nylon membranes (Millipore) consecutively.

### Transfructosylase, Hydrolase, and Inulinase Activity of the Fractions

Hydrolytic and transfructosylating activity evaluated in the four fractions was performed in a 2 mL Eppendorf tube, 900 μL of sucrose solution (4% in acetate buffer, pH 4.5, 50 mM) were deposited, and 100 mL of the enzymatic extract fraction was added. For inulinase activity, in a 2 mL Eppendorf tube 900 μL of inulin solution (1% in acetate buffer, pH 4.5, 50 mM) were deposited and 100 μL of the enzymatic extract fraction was added. The reactions were performed for 20 min at 30°C. The reaction was stopped at 100°C for 3 min. The three enzymatic activities of each fraction were analyzed in HPLC following the methodology described above (section Evaluation and Selection of the Crude Enzymatic Extract). One enzymatic unit of hydrolase (Uh) was determined as the enzyme necessary to liberate 1 μmol of glucose per minute; one enzymatic unit of transfructosylase (Ut) was determined as the enzyme necessary to produce 1 μmol of kestose per minute; and one enzymatic unit of inulinase (Ui) was determined as the enzyme necessary to liberate 1 μmol of kestose per minute, all under certain conditions (10, 17). Protein of every fraction was measured following Bradford's method (18). The selection of the fraction was determined by the highest activity in FOS production.

### Electrophoresis of the Fractions

The selected fraction was analyzed by electrophoresis. It was performed in a miniProtean® Tetra-cell chamber. Twenty microliters of sample were added to the wells and 8 μL of the pre-stained marker #1610318. The running buffer was added and the electrophoresis was performed at 80 V for 30 min and later at 120 V for 1 h. The gels were stained with silver staining technique with the Silver BULLit™ kit, following the fabricant instructions.

### Enzymatic Kinetic in Aguamiel

The enzymatic kinetic of the selected fraction was performed in 2 mL Eppendorf tubes with 900 μL of aguamiel, and 100 μL of the selected fraction were added to every tube. The kinetics were performed at 30, 50, and 70°C, and samples were withdrawn at 1, 2, 3, 4, 5, 10, and 15 h of reaction. The reactions were stopped at 100°C for 3 min. The FOS analysis of the samples was performed by HPLC following the methodology described above (section Evaluation and Selection of the Crude Enzymatic Extract) (17).

### Antioxidant Activity

The antioxidant activity of aguamiel before and after enzymatic treatment was performed by three methods: DPPH and FRAP assays. Antioxidant capacity was determined by a standard curve prepared with a Trolox standard.

#### DPPH Radical Scavenging Activity

The DPPH· assay was carried out according to the methodology reported by Molyneux (19). Seven microliters of each sample were placed in a microplate, and 193 μL of 60 μM DPPH radical solution were added. The microplate was placed in the dark for 30 min and the absorbance was measured at 517 nm.

#### FRAP Assay

Ferric ion reducing antioxidant power assay was carried out according to the methodology reported by Benzie and Strain (20). A 10 mM TPTZ in 40 mM HCl solution was mixed with a 20 mM FeCl_3_ solution and 0.3 M sodium acetate buffer in 1:1:10 (v/v/v) proportions. Ten microliters of each sample were placed in a microplate and 290 μL of the mixture solution were added to each sample. The reaction was placed in the dark for 15 min and the absorbance was measured at 593 nm.

### Prebiotic Activity of Aguamiel Before and After Enzymatic Treatment

Prebiotic activity was performed over *Lactobacillus plantarum* 14917*, Lactobacillus paracasei* 25302*, Bifidobacterium lactis*, and *Bifidobacterium bifidum* 450B, all strains obtained from the culture collection of the Food Research Department of Autonomous University of Coahuila (Mexico). All the strains were previously activated in MRS media culture at 37°C for 24 h. The kinetic growth of the strains was performed in a sterile 96 wells microplate. Aguamiel threated with the enzymatic fraction extract, crude aguamiel, and MRS media culture as a control were added to the microplate. Each strain was added to the three-different media to a final volume of 200 μL per well with a concentration of 1.5 × 10^8^ colony forming units of bacteria. All kinetics were monitored each hour for 24 h spectrophotometrically by recording the OD_600_ variations at 37°C (21). All kinetics were performed by triplicate.

### Statistical Analysis

All experiments were carried out by triplicate. All data were analyzed by a comparison of means. The variance analysis was performed in the Statistica 7 software using the Tukey's range procedure.

## Results

### Crude Enzymatic Extracts Evaluation

The application of the 40 crude extracts showed that only six of them produced FOS using aguamiel as a substrate. All extracts obtained from the fermentation with Orafti HSI® as carbon source at both concentrations (60 and 120 g/L) did not show FOS production, as well as the extracts obtained using aguamiel and agavins as a carbon source between 0 to 18 h of fermentation. In Table 1, the extracts that produced FOS using aguamiel as a substrate are shown. Four of these six extracts were obtained from fermentation with agavins and the other two from aguamiel; the production of FOS was higher with the extracts obtained with agavins at a high concentration (120 g/L) as a carbon source. Furthermore, according to results, the extract obtained with agavins at 120 g/L and 48 h of fermentation showed the highest FOS production (2.26 ± 0.07 g/L). Thus, this extract was selected for further steps.

**Table 1:** Evaluation of enzymatic extracts for FOS production in aguamiel.

**Carbon source**	**Crude extract used for FOS production (g/L)**			
	**24 h**	**30 h**	**36 h**	**48 h**
Agavins (60 g/L)	NP	NP	0.14 ± 0.02	0.11 ± 0.02
Agavins (120 g/L)	NP	NP	1.99 ± 0.11	2.26 ± 0.07
Aguamiel	0.13 ±0.02	1.04 ±0.12	NP	NP

*Orafti HSI® results are not included because these extracts did not show FOS production at any of evaluated times*.

### Fractioning and Enzymatic Activities

**Figure 1:**
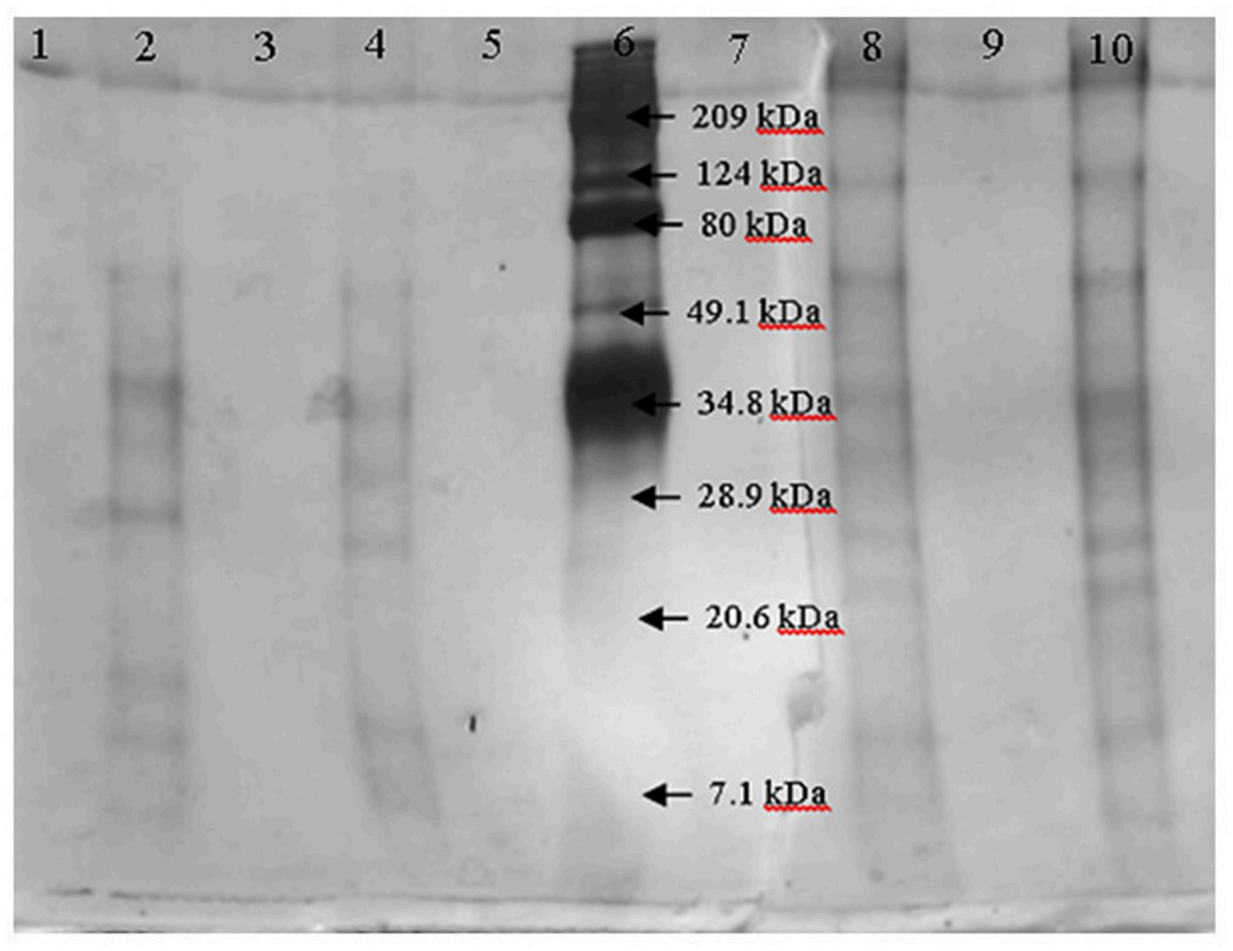
Electrophoresis of the fractions < 30 kDa (lanes 2 and 4) and 30–100 kDa (lanes 8 and 10) by duplicate. Marker is in lane 6.

After fractioning the selected crude extract, the three activities measured showed different performances. In Table 2, all the enzymatic activities of the four fractions are shown. Transferase activity was present in the four fractions, but the highest activity was shown in the 30–100 kDa fraction, with an activity almost five times greater than that of the other fractions. Hydrolytic activity was only present in the lower size fractions and the inulinase activity was only present in the < 30 kDa fraction. Due to the enzymatic activities shown by the four fractions, the 30–100 kDa fraction was selected to be analyzed by electrophoresis and the < 30 kDa fraction was analyzed to compare both fractions and analyze which protein could be responsible for inulinase activity (present only in < 30 kDa fraction). In Figure 1, the electrophoretic profile of both fractions is shown; the fraction < 30 kDa depicted four different proteins with molecular weights of ~7, ~11, ~29.5, and ~34 kDa. The 30–100 kDa fraction showed proteins with molecular weights of ~23, ~26.5, ~32, ~35, ~65, and ~124 kDa.

**Table 2:** Enzymatic and specific activity of the four different fractions.

**Fraction**	**Enzymatic activity**			**Specific activity**		
	**Hydrolytic (Uh/L)**	**Transferase (Ut/L)**	**Inulinase (Ui/L)**	**Hydrolytic (Uh/mg)**	**Transferase (Ut/mg)**	**Inulinase (Ui/mg)**
300–450 kDa	0	309.20	0	0	77.11	0
100–300 kDa	0	299.72	0	0	74.74	0
30–100 kDa	1799.79	1473.15	0	488.81	367.39	0
< 30 kDa	699.45	220.07	443.64	367.36	54.88	110.63

### FOS Production

**Figure 2:**
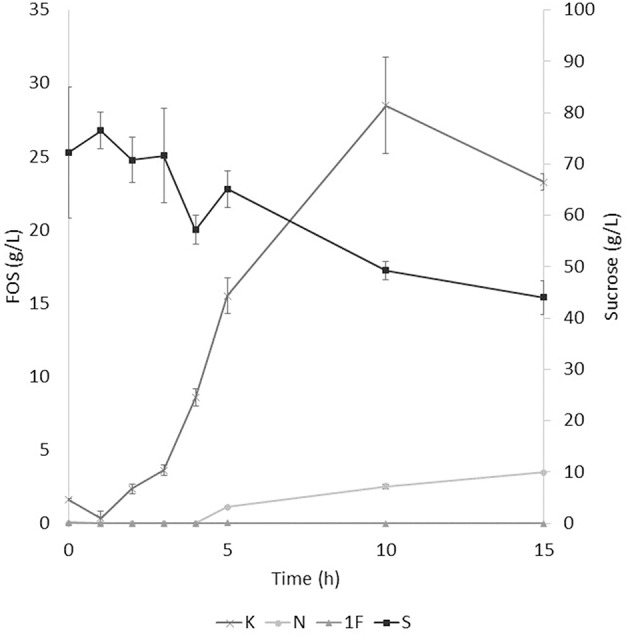
Enzymatic kinetic of the selected fraction over aguamiel carried out at 30°C. K, Kestose; N, Nystose; 1F, 1-β-fructofuranosylnystose; S, Sucrose.

**Figure 3:**
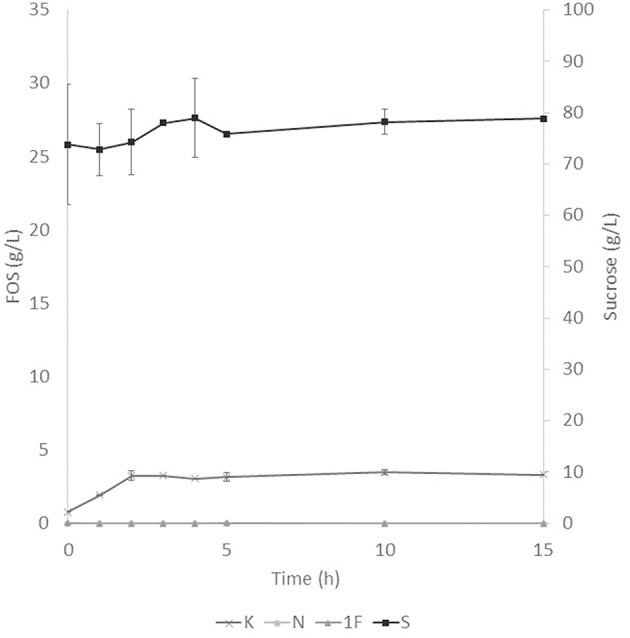
Enzymatic kinetic of the selected fraction over aguamiel carried out at 50°C. K, Kestose; N, Nystose; 1F, 1-β-fructofuranosylnystose; S, Sucrose.

**Figure 4:**
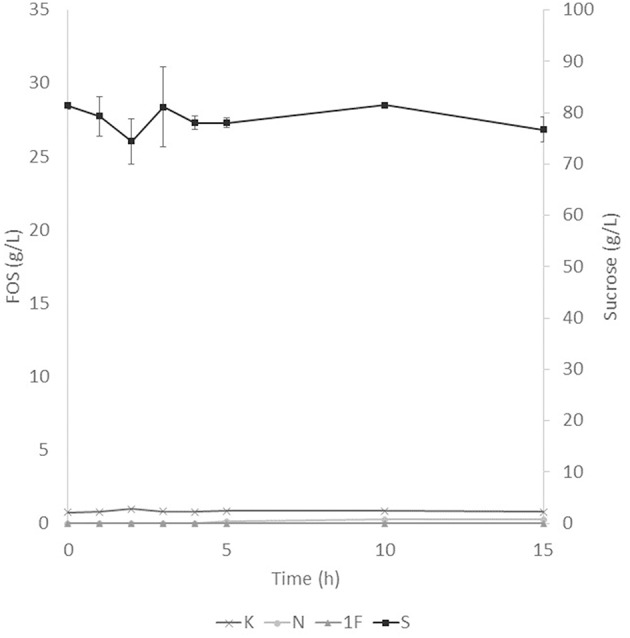
Enzymatic kinetic of the selected fraction over aguamiel carried out at 70°C. K, Kestose; N, Nystose; 1F, 1-β-fructofuranosylnystose; S, Sucrose.

The application of the selected fraction over aguamiel showed better results in the 30°C kinetic (Figure 2). The 30°C kinetic showed the formation of FOS along the 15 h of the kinetic, but the highest concentration of FOS was achieved at 10 h with 31.01 ± 3.42 g/L total FOS concentration in aguamiel. In comparison with the initial concentration of 1.61 ± 0.08 g/L, it is almost 20-fold the initial concentration of FOS. Also, the kinetic depicted the standard FOS formation by transfructosylating activity, developing the formation of kestose and then the formation of nystose. The 50°C kinetic showed an increase in kestose concentration in the first 2 h (Figure 3), but after this time FOS concentration remained constant, without increasing any of the three FOS measured. Kinetic at 70°C did not show results or changes within the 15 h of the reaction (Figure 4).

### Antioxidant Activity

The antioxidant assay showed a difference between the aguamiel before and after the enzymatic treatment. DPPH and FRAP assays showed higher antioxidant activity on aguamiel after the enzymatic treatment; the difference was three times the antioxidant activity compared to the aguamiel without treatment in DPPH assay, and over five times in the FRAP assay, as shown in Table 3.

**Table 3:** Antioxidant analysis of aguamiel without enzymatic treatment and aguamiel after the enzymatic treatment with the high FOS concentration.

**Sample**	**Antioxidant assay (mg/mg eq Trolox)**	
	**DPPH**	**FRAP**
Aguamiel before enzymatic treatment	34.81 ± 5.75	42.65 ± 5.85
Aguamiel after enzymatic treatment	116.46 ± 0.48	298.86 ±26.76

### Prebiotic Activity

**Figure 5:**
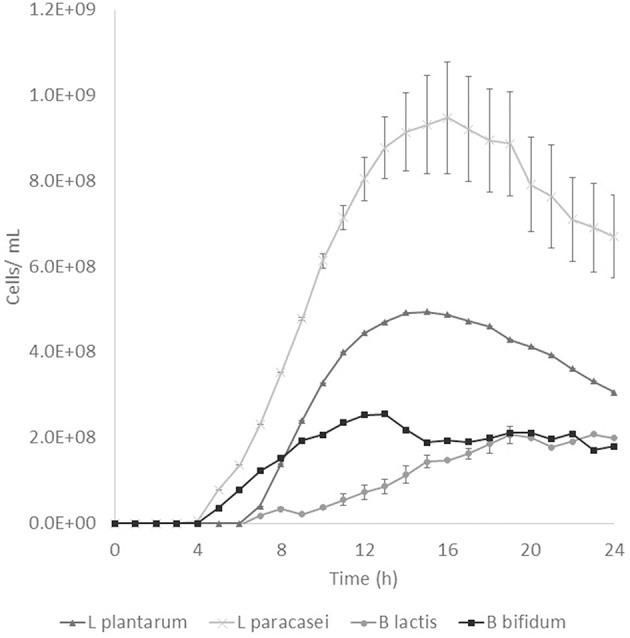
Probiotic bacteria growth in aguamiel. Bacterial growth in aguamiel after enzymatic treatment with high FOS concentration.

**Figure 6:**
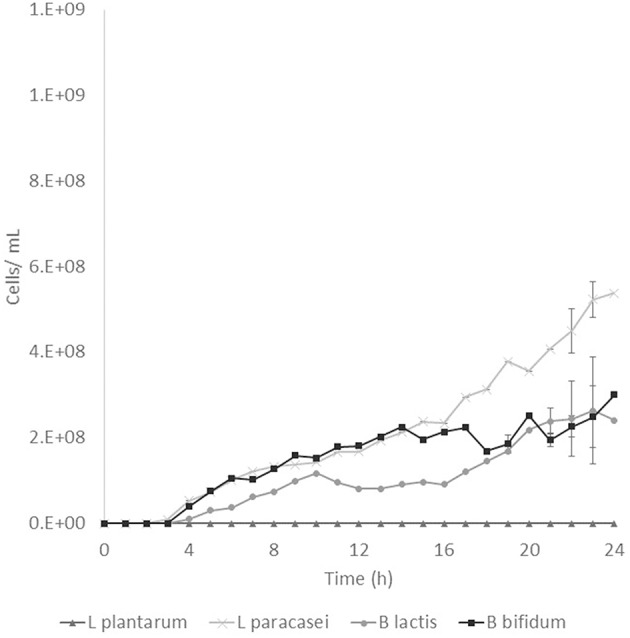
Probiotic bacteria growth in aguamiel. Bacterial growth in aguamiel without enzymatic treatment.

Prebiotic activity showed different results between treated (T) and non-treated aguamiel (NT). The *Lactobacillus* species showed a faster growth over the aguamiel with a high concentration of FOS (T). In Figure 5, faster growth of both *Lactobacillus* species can be observed, both species having their maximum growth between 12 and 16 h, compared with the same species evaluated over NT aguamiel (Figure 6) where *L. plantarum* 14917 did not grow and *L. paracasei* 25302 had its maximum growth at 24 h (almost half of the maximum growth of this bacteria in aguamiel with a high concentration of FOS). *B. bifidum* 450B showed a faster growth over the treated aguamiel, reaching its higher growth at 13 h, which was two times faster than the growth of 2.1 × 10^8^ cells/ mL over the non-treated aguamiel; in the non-treated aguamiel *B. bifidum* 450B reached its maximum growth of 2.2 × 10^8^ cells/mL at 24 h. *B. lactis* showed a higher growth over the non-treated aguamiel, the maximum growth of this bacteria was almost the double the maximum growth in the treated aguamiel.

## Discussion

### Crude Enzymatic Extracts Evaluation

The evaluation of the extracts over aguamiel showed the influence of the carbon source used to induce the production of FOS producing enzymes. In the case of Orafti HSI®, these extracts showed hydrolytic activity, which increased as the fermentation progressed. Due to this behavior, the application of these extracts was discarded to the purpose of this work. Extracts obtained from aguamiel showed FOS production with extracts obtained at 24 and 30 h; this behavior was similar to the activities that Muñiz-Marquez et al. (17) reported, where the maximum fructosyltransferase activity was between 24 and 32 h of fermentation using *A. oryzae* DIA-MF. The extracts obtained from agavins fermentation showed FOS production at longer times (36 and 48 h) compared to that obtained from aguamiel, and the best FOS producer was the extract obtained at 48 h of fermentation at high-level agavins concentration. Other authors reported the production of the enzymatic extracts at the same times, Guio et al. (22) used *A. oryzae* N74 strain to produce FOS at 48 h of fermentation; Ottoni et al. (23) used *A. oryzae* IPT-301strain to a final fermentation time of 72 h and Kurakake et al. (24) used *A. oryzae* KB at a final fermentation time of 96 h. Comparing the fermentation times to produce the enzymatic extract, the use of agavins or aguamiel to produce FOS producing enzymes at short fermentation times can be advantageous. Also, the concentration of the substrate has an important role in the expression of the enzymes; when comparing the FOS production of the extracts at the same time of fermentation from agavins, the production is over 10 times the production with the extracts from high agavins concentration.

### Fractioning and Enzymatic Activities

After the fractioning of the crude enzyme extract, different proteins were observed in the fractions (< 30 and 30–100 kDa). The presence of inulinase activity in the < 30 kDa fraction and the different proteins on the electrophoresis of this fraction compared to the 30–100 kDa fraction, suggest that one or more of these proteins may be responsible for this activity, but some researchers reported inulinases with low molecular weights for an inulinase of 30 kDa from *Rhizopus oligosporus* or 31 kDa from *Aspergillus ficuum* (25, 26). Also, the production of an inulinase with a lower molecular weight could be happening by *A. oryzae* DIA-MF induced by the agavins.

The higher hydrolytic and transfructosylating activity on the 30–100 kDa fraction can be produced by one or more of the proteins in it. If the responsibility of the enzymatic activity lies with one protein, then the enzyme could be a β-fructofuranosidase due to its high hydrolytic activity or it could be a conjunction of two enzymes; one β-fructofuranosidase and one fructosyltransferase with high hydrolytic and transfructosylating activity. Muñiz-Marquez et al. (17) reported the production of a fructosyltransferase with high transference activity produced by *A. oryzae* DIA-MF and Kurakake et al. (24) reported the production of two β-fructofuranosidases by *A. oryzae* KB, one with high transfructosylating activity and the other one with high hydrolytic activity. The production of only one enzyme with both activities or two different enzymes by the fungi could be possible. Spohner and Czermak (27) reported a fructosyltransferase with a molecular weight of 80 kDa approximately, Fernandes et al. (28) reported a β-fructofuranosidase with a molecular weight of 37 kDa similar to the Lincoln and More (29) report of a β-frutofuranosidase of 35 kDa, both from an *Aspergillus* species, and Kurakake et al. (24) reported the two β-fructofuranosidases of 96 and 79 kDa. The activity of more than one enzyme in the 30–100 kDa fraction with one or two activities could be occurring due to the presence of six different proteins.

### FOS Production

After the three temperature treatments in the enzymatic kinetic, the 70°C treatment did not show an increment in FOS and the 50°C showed an increment only at 2 h. This could be due to the low thermostability of the enzyme, at 70°C the proteins began to denaturalize immediately and at 50°C they were denaturalized but at a lower speed. The proteins could tolerate the 50°C for a short time but when applying longer times it begins to denaturalize; instead FOS production by enzymes with fructosylating activity showed their optimal temperatures between 50 and 60°C (8). According to this behavior in both treatments, we can conclude that the enzymes produced were not thermostable. The treatment of 30°C started producing kestose from the sucrose that aguamiel contains, as it is shown in Figure 2. The FOS production begins with kestose, and the formation of nystose was next; this mechanism is the main mechanism that a transfructosylating enzyme uses to produce FOS from sucrose (8). In accordance with the mechanism of FOS formation, the formation of fructooligosaccharides with higher DP could take more time, the production of nystose is slower than kestose, and the concentration of kestose begins to decrease as it can be observed after 10 h of the enzymatic reaction, kestose is reduced in concentration as nystose continue increasing. The production of 1-β-fructofuranosylnystose was not possible at the times the kinetic was carried out.

### Antioxidant Activity

FOS have been reported with antioxidant activity, Manosroi et al. (30) reported FOS extracted from *Coix lachryma-jobi* Linn to have an antioxidant activity similar to Vitamin C; Zhang et al. (31) evaluated the antioxidant capacity in fish fed with a low FOS concentration diet, and the results showed the increment of liver catalase and superoxide dismutase activities. The antioxidant activity was similar in both assays but not within treatments. Antioxidant activity from aguamiel after the enzymatic treatment was higher (116.46 ± 0.48 and 298.86 ± 26.76 mg/mg Eq Trolox for DPPH and FRAP, respectively), and in both cases the activity was 4 times better than the activity from the non-treated aguamiel (Table 3). Higher antioxidant activity could be related to the partial enzymatic degradation used to produce FOS or other fructose compounds could induce the formation of new antioxidants, such as heterocyclic compounds (5). Enzymatic hydrolysis of inulins and agavins could release FOS, increasing the content of terminal fructoses with reducing capacity able to participate in antioxidative reactions. Mesa et al. (32) also suggested that FOS with molecular masses lower or equal to 10 kDa are an important source of antioxidants that are able to scavenge peroxyl radicals and to prevent *in vivo* LDL oxidation.

### Prebiotic Activity

FOS are compounds known as prebiotics due to their ability to reach the intestine microbiota and be used as a substrate by microbiota. It is known that *Lactobacillus* and *Bifidobacterium* are the two bacterial groups which can metabolize oligosaccharides, and both are part of the intestinal microbiota (4). The application of aguamiel with a high FOS concentration showed a different growth on the four bacteria used. A difference was observed in the growth pattern of the two *Lactobacillus* strains used. *L. plantarum* 14917 and *L. paracasei* 25302 had 6.4 × 10^7^ and 9.1 × 10^7^ cell/mL/h specific growth rates, respectively, in aguamiel after the enzymatic treatment. Meanwhile, the specific growth rate on the non-treated aguamiel was 2.5 × 10^7^ cell/mL/h for *L. paracasei* 25302; *L. plantarum* 14917 did not grow in this aguamiel. In addition, there was a significant statistical difference in the growth between the *Lactobacillus* strains. On the other hand, *Bifidobacterium* bacteria growth has similar behavior, the specific growth rate of *B. lactis* and *B. bifidum* 450B in the treated aguamiel was 1.5 × 10^7^ and 3.1 × 10^7^ cell/mL/h, respectively, compared to the growth on the non-treated aguamiel which was 1.3 × 10^7^ and 5.3 × 10^6^ cell/mL/h. *B. lactis* had similar growth on both aguamiel, whereas, *B. bifidum* 450B had the same behavior of the *Lactobacillus* strains, with a higher specific growth rate. Higher growth of probiotic bacteria could be related to the production of β-fructofuranosidase, and its participation in conjunction with the sucrose phosphoenolpyruvate transport system to consume the substrate and provide the enzyme with a better substrate consumption for its growth (33). Due the high sucrose concentration in the non-treated aguamiel, this could be affecting the bacterial growth. The treated aguamiel demonstrated better conditions for improving the probiotic bacterial growth and due to the capacity of FOS to reach the intestinal microbiota, the potential for use of aguamiel as a food rich with prebiotic compounds is high.

Finally, we can conclude that aguamiel treated with the enzymatic fraction showed a high conversion of sucrose to FOS. This conversion indicates an opportunity to use aguamiel as a functional food due to the concentration of FOS, which was increased from 1.61 ± 0.08 to 31.01 ± 3.42 g/L after 10 h reaction at 30°C applying 10% enzymatic fraction-substrate (v/v). Considering the minimum daily effective intake of FOS, a portion of 100 mL of treated aguamiel could promote health benefits, compared to a portion of ~2 L of non-treated aguamiel. Antioxidant activity was highly increased (34.81–116.46 mg/eq Trolox in DPPH assay and 42.65–298.86 mg/eq Trolox in FRAP assay) and growth of probiotic bacteria was higher in aguamiel after the enzymatic treatment. Due to the FOS concentration in the treated aguamiel, this product proved to have higher antioxidant activity and probiotic bacterial growth. After the application of the enzymatic treatment, aguamiel was enriched with FOS which improved antioxidant and prebiotic properties, therefore, it can be used as a functional food.

## Author Contributions

BP executed all the experiments presented in this paper, within the framework of his postgraduate thesis. AF-G conducted the experiments that were presented and supervised the work performed by BP, as his thesis director. AI advised the experiments related to the fractionation of proteins and facilitated the materials to be able to carry it out. RR-J advised BP's work regarding the quantification of sugars present in aguamiel. CA participated in the co-direction of the work and in conjunction with AF-G and BP, designed the experiments and supervised the execution of the same.

### Conflict of Interest Statement

The authors declare that the research was conducted in the absence of any commercial or financial relationships that could be construed as a potential conflict of interest.
